# Information-Based Social Coordination Between Players of Different Skill in Doubles Pong

**DOI:** 10.3389/fpsyg.2018.01731

**Published:** 2018-09-19

**Authors:** A. A. M. (Daphne) van Opstal, Niek H. Benerink, Frank T. J. M. Zaal, Remy Casanova, Reinoud J. Bootsma

**Affiliations:** ^1^Center for Human Movement Sciences, University Medical Center Groningen, University of Groningen, Groningen, Netherlands; ^2^Institut des Sciences du Mouvement, Aix-Marseille Université, CNRS, Marseille, France

**Keywords:** social coordination, visual information, interception, team performance, skill level, emergent behavior

## Abstract

We studied how teams of two players of different skill level intercepted approaching balls in the doubles-pong task. In this task, the two players moved their on-screen paddles along a shared interception axis, so that the approaching ball was intercepted by one of the paddles and that the paddles did not collide. Earlier work revealed the presence of a fuzzy division of interception space, with a boundary between interception domains located in the space between the two initial paddle positions. In the present study, using the performance of the players in their individual training sessions, we formed teams of players of varying skill level. We considered two accounts of how this boundary should be understood. In a first account, the players have shared knowledge of this boundary. Based on the side of the boundary at which the approaching ball will cross the interception axis, the players would decide whose paddle is to make the interception. Under this account, we expected that a better-skilled player would take responsibility for a larger interception domain, leading to a boundary closer to the lesser-skilled player. However, our analyses did not reveal any systematic effect of skill difference on the location (or degree of fuzziness) of the boundary: location of boundaries and overlap of interception domains varied over teams but were not systematically related to skill differences between team members. We did find effects of ball speed and approach angle. In a second account, the boundary emerges from (information-driven) player–player–ball interactions. An action-based model consistent with this account was able to capture all the patterns in boundary positions and overlaps that we observed. We conclude that the interception patterns that players demonstrate in the doubles-pong task are best understood as emerging from the unfolding of the dynamics of the system of the two players and the ball, coupled through information.

## Introduction

Team work implies coordination. Teams are made of individuals, and individuals differ. How do these differences play out in the coordination among the team members and their environment? Consider, for instance, the situation in which a group of friends helps to move furniture to a new apartment. To carry a sofa up the stairs, at least two people are needed. Best practice learns that the stronger person of the two best carries more weight standing under the sofa and the other person carries less weight but also guides the sofa’s movement through the stairwell. Such a division of labor can be planned and communication throughout the operation facilitates the coordination (e.g., Vesper et al., 2017). In professional or sports situations, teams are often composed of specialists who work together on a shared goal. For instance, Gray et al. (2017) studied how teams of baseball infielders coordinated their actions in response to balls hit to the infield. Being at their own position (i.e., the position that they were used to play at) with teammates (i.e., players whose action capabilities they knew best) led to the most successful team decisions. In this situation, team decisions had to be made quickly and some predictability from experience with teammates was beneficial (cf. Glover and Dixon, 2017). Even clearer differences in expertise can be found in teams flying drones for reconnaissance purposes. Cooke and colleagues studied teams composed of a pilot, a navigator, and a photographer, who collaborated in flying simulated drone missions in order to take photographs of reconnaissance targets (for an overview, see Cooke et al., 2013). These studies demonstrated that to understand how successful team decisions come about, a good understanding of the interactions among the team members is indispensable. For instance, when comparing different types of training, Gorman et al. (2010) demonstrated that teams that had received a training focused on interactions among the team members were better able to adapt to novel situations that asked for performance under increased workload. Thus, interaction among team members seems key to success. Team members often have different roles, each contributing to the shared goal. But what if team members have the same role but different abilities?

The current study builds on previous work on joint interception, with teams of two individuals performing a doubles-pong task (Benerink et al., 2016, 2018). An innovative aspect of these studies was that team members were not assigned specific roles as to who was supposed to intercept balls where. In the doubles-pong task, each team member controlled their own paddle that could be moved along an interception axis at the bottom of a large, shared computer screen. Starting from different positions, balls moved along rectilinear paths from the top of the screen downward, under different angles with the vertical. With overt communication being banned, the task of the team on each trial was simply to intercept the ball. Importantly, the task constraints dictated that successful interception could in fact only be accomplished with a single paddle, as contact between the paddles led both to immediately disintegrate rendering future interception impossible. Inspection of how teams dealt with this joint-interception task revealed that they systematically showed a division of interception space. There was a distinct boundary between the interception domains of both players, together with a fair amount of overlap. When considering the teams in the Benerink et al. (2016) study, this boundary was generally located roughly halfway between the two paddles’ initial positions, although some inter-team variability in its location was present. One notable exception in this study was a team with a boundary between interception domains clearly located away from the middle. Particularly interesting for the present purposes was that this specific team was characterized by a considerable difference in the individual skill levels of its two members and that the boundary was shifted toward the lesser-skilled player’s initial paddle position. In other words, it seemed that the better-skilled player had taken responsibility of a larger interception domain. Moreover, a pilot experiment, in which we had teams perform the doubles-pong task while both players operated paddles of a different size, accidentally included skill differences between team members. Here too, these skill differences seemed to affect the location of the boundary, such that the boundary was closer to the lesser-skilled player at a distance from the mid-screen vertical that seemed linearly related to the skill difference (Benerink et al., 2015). The current study was inspired by these findings and set out to explore the question how joint interception plays out when team members differ in skill level on the same task.

The boundaries observed in the Benerink et al. (2016, 2018) studies bring to mind the boundaries in so-called Voronoi diagrams (e.g., Rein et al., 2017) or dominant regions (Taki and Hasegawa, 2000), as applied in a number of team-sport situations. When considering soccer, for instance, the pitch can be tessellated into areas such that each area is comprised of all positions on the field closest to the player occupying that area. Boundaries between these areas are lines halfway between adjacent player positions. Such spatial tessellations (i.e., Voronoi diagrams) have been applied in soccer (Taki and Hasegawa, 2000; Kim, 2004; Rein et al., 2017), futsal (Fonseca et al., 2012), volleyball (Paulo et al., 2018), and handball (Taki and Hasegawa, 2000), for example, in relation with passing opportunities (Gudmundsson and Wolle, 2014). Interestingly, one of the earlier studies that sought to apply the Voronoi diagrams took the tessellation in a direction that is directly relevant for the current study. Taki and Hasegawa (2000) suggested that the determination of the boundaries between Voronoi cells should not be limited to purely geometrical considerations but should also include the speed that players can adopt in all different directions. Indeed, in the same time, a player can reach a larger distance running in the forward direction than running in the backward direction. Taking into account the players’ orientations, asymmetries can thus appear when drawing the Voronoi diagrams. Analogously, when looking at boundaries between two players who differ in the maximum speed they are able to reach, an asymmetry better captures the situation. In other words, the boundaries would be drawn based on the action capabilities of the players rather than simply on the geometry of the distribution of the players across the pitch.

While, evidently, boundaries can be drawn between the interception domains in the doubles-pong task and other team-based sport examples (using Voronoi diagrams or other methods), the status of such boundaries, however, remains unclear. In one account, the boundaries form the *a priori* basis for team coordination; alternatively, the boundaries are but the *a posteriori* result (by-product) of team coordination dynamics (cf. Benerink et al., 2016, 2018). Returning to the doubles-pong task, successful coordination implies a successful interception by one, and only one, of the two players. In the first-mentioned account, the decision that the players make – about intercepting an approaching ball or leaving that to the teammate – would be based on whether or not the ball will pass the interception axis either on their side of the boundary or on the teammate’s side. Since in our doubles-pong task overt communication between players was banned, such an account would assume that the two players tacitly agreed on the location of the boundary. Furthermore, such an account would (have to) assume that, at some point, players are able to accurately know where a ball will pass the interception axis. In other words, the decision of each player to either intercept or forfeit would then be understood as resulting from their shared understanding of a separation of interception domains (see Vesper et al., 2017 for a review on the role of shared knowledge in joint action) combined with their sufficiently accurate individual predictions of the future ball arrival position. Yet, current models of the control of interception cast doubt on proficiency of performing such predictions. Rather than relying on predictive control (i.e., predict the interception location from early target kinematics and move to this location), interception has been found to be controlled prospectively (i.e., through continuous guidance of the hand to the interception location on the basis of prospective information, e.g., Peper et al., 1994; Montagne et al., 1999; Dessing et al., 2002; Michaels et al., 2006; Ledouit et al., 2013).

**FIGURE 1:**
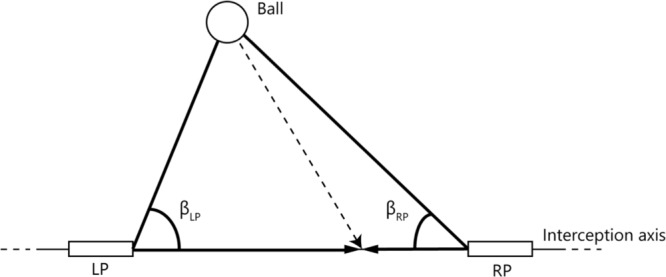
Definition of angles used to capture the relations between the paddles and the ball. LP and RP represent the paddles of the left and right participant, respectively, that could freely move along the interception axis. β_LP_ and β_RP_ are the angles formed by the line connecting both paddles and the lines connecting each paddle with the ball.

An alternative to the account of reliance on an *a priori* boundary that delineates interception domains is one in which the boundary emerges from the unfolding of the dynamics of team coordination (see Richardson et al., 2015; Nalepka et al., 2017). Both players not only see the ball, but also their own paddle and the paddle of their teammate. Benerink et al. (2016) suggested that the division of labor between the two players emerges from the informational couplings within this tripartite system. This account thus focuses on the interactions rather than on the individuals (cf. Cooke et al., 2013). The relevant information is captured by the rates of change of the base angles β (see **Figure 1**) of the triangle formed by the ball (apex) and the two paddles that can move along the horizontal interception axis (base). When either the ball and/or one (or both) paddle(s) move, the relevant angles change. However, in the situation that paddle and ball movement are coordinated in such a way that the corresponding base angle β remains constant (i.e., *d*β/*dt* = 0), ball-paddle contact is forthcoming (e.g., Fajen and Warren, 2007; Bootsma et al., 2016). Benerink et al. (2016, 2018) showed that, for balls heading for positions located between the players’ initial paddle positions, both players often started to move and that attributing interception to the first player whose paddle moved such that its *d*β/*dt* reached (or, in fact, exceeded) zero captured the division of interception space very well. In other words, when interception was afforded to one player, the teammate abandoned his or her movement, to avoid collision and thus allow successful team performance. The latter account assumes that players not only are able to see the affordance of interceptability for themselves (Postma et al., 2018) but also for the other (e.g., Stoffregen et al., 1999; Ramenzoni et al., 2008; Fajen et al., 2009; Weast et al., 2011). Note that in this account the boundaries between interception domains (*a posteriori*) describe the patterns resulting from the unfolding dynamics but do not form the (*a priori*) basis for these patterns.

With the exception of the one team mentioned before, Benerink et al. (2016, 2018) only considered teams with players of similar skill level. In both studies, each player started the experiment with an individual session, not only serving as training on the interception task and apparatus, but also allowing individual skill levels to be determined. Teams were then composed for subsequent doubles sessions by combining players of comparable skill levels (i.e., having similar performance scores) in their individual sessions. The one-team exception herein in the Benerink et al. (2016) study resulted from having to combine a limited pool of 12 players into six teams. In order to investigate effects of skill-level differences between the two team members, in the present study, we deliberately composed teams of players having demonstrated different levels of performance in the preliminary individual sessions. If teams were to rely on a (tacitly) shared understanding of a boundary separating interception domains, balls moving toward the left side of the boundary would be for the left player to intercept and vice versa for the right player. In order to optimize team performance, under this logic, the boundary could then be expected to be shifted toward the lesser-skilled player, with the better-skilled player thereby taking responsibility for a larger interception domain. An account of emergent boundaries, on the other hand, does not necessarily lead to specific predictions concerning the location of the boundary as a function of the skill differences, since it is based on the way players move during an interception attempt, rather than on final outcome. Of course, skill differences might be accompanied by differences in movement kinematics and the interactions between players would then play out into one player intercepting balls at certain locations on the interception axis rather than the other. However this may be the account of emergent division of labor would under all circumstances predict that observed patterns in boundary and overlap can be captured by the model that attributes the interception to the player whose paddle moves such that its associated *d*β/*dt* first reaches zero.

## Materials and Methods

### Participants

In the framework of the present study, participants took part in three separate sessions: one individual session and two doubles sessions. A group of 28 right-handed (post)graduate students from the Aix-Marseille University (17 men and 11 women, with an average age of 24.7 ± 2.2 years, *M ± SD*) volunteered for participation in the first (individual) session. From this group of 28 participants, 12 (eight men and four women, with an average age of 24.8 ± 1.2 years) were retained for the present purposes; the other 16 participated in a separate study (cf. Benerink et al., 2018). The selection of participants for the present study was based on their levels of performance in the individual session, allowing teams (i.e., dyads) with different individual performance levels to be composed (details follow later).

All participants provided written consent before participating in the study that was approved by the local institutional review board of the Institute of Movement Sciences (Comité Ethique de l’Institut des Sciences du Mouvement d’Aix-Marseille Université) and conducted according to University regulations and the Declaration of Helsinki.

### Experimental Setup

The experimental setup used for the present experiment was the same as that of Benerink et al. (2016). The experiments were all performed in a darkened room equipped with a large table with two adjacent seats on one side and a large television screen (Samsung 55” LED ED55C, operating at a frame rate of 100 Hz with a 1920 × 1080 pixel resolution) on the other side. Seated participants faced the middle of the screen at eye-height from a 2-m distance. Participants were separated by a curtain, hanging down from the ceiling, that prevented them from seeing (any part of) the other during the doubles sessions. With verbal communication between participants being banned, headphones (3M Peltor Optime2) and earplugs furthermore prevented them from picking up (auditory) information about their partner’s behavior.

**FIGURE 2:**
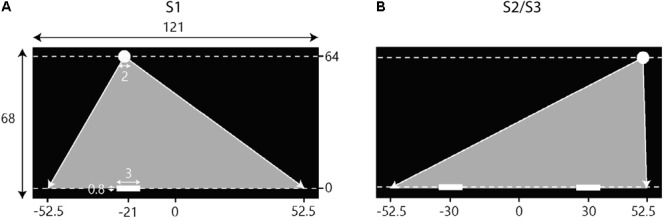
Schematic overview of the setup of the experimental sessions. Screen dimensions and other metrics are in cm. Note that the figures are not scaled to actual size. Balls appeared at the top of the screen (*Y* = 64) and moved downward toward the interceptions axis (*Y* = 0) at one of two constant vertical velocities. Gray triangles indicate the range of potential ball arrival positions. **(A)** During the first session (S1) participants intercepted balls individually. The situation depicted here represents the initial conditions for a left position player. **(B)** In the second (S2) and third (S3) sessions, participants intercepted balls in dyads where on player started on the left side of the screen and the other player started on the right side of the screen.

Participants individually controlled the position of their on-screen paddle by moving a hand-held knob laterally over an in-house constructed linear-positioning device placed on the table in front of them (for further details, see Benerink et al., 2016). The on-screen paddle moved along the (invisible) horizontal interception axis, located just above the bottom of the screen that extended horizontally (*X*-axis) from -60.5 to +60.5 cm and vertically (*Y*-axis) from -2 to +66 cm (see **Figure 2**). A proportional gain ensured that participants could cover the full (121-cm) range of the on-screen interception axis with their paddle without reaching the extremities of the (75-cm long) linear-positioning device. Unless specified otherwise, positions and distances reported from here on correspond to distances on the screen, with the origin corresponding to the center of the horizontal interception axis.

Positions of the participants’ paddles and the ball were sampled at a frequency of 100 Hz and stored on an external disk. Prior to analysis, the kinematic data were filtered with a recursive low-pass second-order Butterworth filter with a cut-off frequency of 5 Hz (cf. Benerink et al., 2016, 2018).

### Task and Procedure

The participants’ task was to intercept virtual balls (2-cm diameter white circles depicted against a black background), moving downward across the screen at various angles and speeds, by making these bounce back upward after contact with a white (3-cm wide and 0.8-cm high) paddle.

The first session (S1), in which participants performed the interception task individually, consisted of five blocks of 50 trials, for a total of 250 trials per participant. In this session, half of the participants performed the task while seated on the left side of the table (left position) and the other half performed the task while seated on the right side of the table (right position). As reported in Benerink et al. (2016), performance generally increased over the first two blocks before leveling off on the last three blocks. The skill level demonstrated in S1 by each participant was therefore operationally defined by score S, calculated as S = (B3 + B4 + B5 + Max/2)/3.5, where B3, B4, and B5 correspond to the percentage of balls intercepted in blocks 3, 4, and 5 and Max corresponds to the highest percentage of balls intercepted in any of the five blocks.

Individual S-scores (see **Table 1**) were used to form teams composed of individuals with different skill levels for the doubles sessions. Since pilot work indicated that participants that took part in an individual session in either the left or the right position subsequently performed equally in both positions, team composition for sessions 2 (S2) and 3 (S3) did not take into account individual participant positions in S1. However, all participants performing in the left position in S2 performed in the right position in S3 (hereafter referred to as P1 participants). Likewise, all participants performing in the right position in S2 performed in the left position in S3 (hereafter referred to as P2 participants). In order to test the basic hypothesis of a shift in boundary location (toward the less-skilled player) in the presence of within-team skill-level differences, in S2, the 12 participants were combined into six teams with relatively homogeneous differences in S-scores within teams, ranging from 6.3 to 9.4% (*M* ±*SD* = 7.7 ± 1.2%). In order to test the hypothesis that the shift in boundary location varied as a function of the degree of within-team skill-level differences, for S3, six new teams were formed, with differences in S-scores within teams now varying from 2.3 to 13.4% (*M* ±*SD* = 6.4 ± 5.3%). While perhaps seemingly moderate, these skill-level differences between team members (see **Table 2** for details) are to be appreciated in the light of the 1.8 ± 1.5% (range 0.4–4.8%) and 1.7 ± 0.8% (range 1.0–2.5%) within-team differences in individual performance for, respectively, all eight teams of the Benerink et al. (2018) study and five of the six teams of the Benerink et al. (2016) study^1^. Over the two doubles sessions of the present experiment, within-team differences in S-scores were on the average 7.1 ± 3.7%.

**Table 1:** Individual characteristics for session 1 (S1) of the 12 participants.

Participant	Pos S1	Perf (%)	S (%)
A	L	86.4	90.0
B	R	95.2	97.1
C	L	79.2	84.3
D	R	88.0	92.3
E	R	94.4	96.3
F	L	84.0	90.0
G	R	87.2	92.3
H	R	82.8	82.9
I	L	80.0	86.3
J	L	90.0	92.9
K	L	86.8	90.0
L	R	78.8	81.4
	Mean	86.1	89.7
	SD	5.4	5.0

*Pos S1, S1 in left (L) or right (R) position; Perf, performance expressed as the mean percentage balls intercepted over all five blocks of S1; S, skill-level score used for composing teams.*

**Table 2:** Team characteristics and results.

Session	Team	P1	P2	P2–P1 (%)	TP (%)	B-Loc (cm)	Overlap (cm)
S2	9	A	B	7.1	94.0	3.0	13.0
S2	10	C	D	8.0	88.5	3.3	10.3
S2	11	E	F	–6.3	86.5	–1.4	11.7
S2	12	G	H	–9.4	84.5	3.9	24.8
S2	13	I	J	6.6	89.5	–2.8	10.2
S2	14	K	L	–8.6	85.5	2.6	16.3
S3	15	A	D	2.3	92.0	0.2	12.3
S3	16	C	B	12.8	87.5	1.4	11.0
S3	17	E	H	–13.4	90.0	3.4	14.1
S3	18	G	F	–2.3	85.0	3.4	19.2
S3	19	I	L	–4.9	76.0	3.7	13.2
S3	20	K	J	2.9	88.5	0.4	22.8
			Mean	| 7.1|	87.3	1.8	14.9
			SD	3.7	4.3	2.2	4.9

*P2–P1, within-team S-score difference between P2 and P1 participants; TP, team performance (% intercepted); B-Loc, boundary location between interception domains; overlap, overlap between interception domains.*

In both doubles sessions, participants were instructed that the task they had to perform was to intercept as many balls as possible as a team by moving the on-screen paddles laterally along the invisible horizontal interception axis. Importantly, participants were warned that they should avoid contact between their on-screen paddles, as this led both paddles to immediately disintegrate, thereby rendering future interception impossible. Participants were explicitly instructed that the number of individual interceptions did not matter and that the team performance was the only thing that counted.

For a trial to start, participants had to move their paddle to the designated start position (30 cm to the left or to the right of the center of the screen in S2 and S3; see **Figure 2**) marked by a 3-cm wide translucent red rectangle. If the center of the participant’s paddle arrived within 0.3 cm of the center of the rectangle, the rectangle turned green indicating that the paddle was located at the right place. After participants had remained in place for 2 s, the green rectangles disappeared and after another second the ball appeared. Balls moved downward with vertical speeds of 0.40 [slow ball speed (BS)] or 0.64 m/s (fast BS), corresponding to movement durations for the ball to arrive at the interception axis of 1.6 and 1.0 s, respectively. Successful interception required that one of the participants’ paddles touched the ball before it crossed the interception axis. If so, both paddles turned green and the ball moved back up again. In trials in which neither of the two participants reached the arrival position of the ball in time (i.e., unsuccessful trials), the paddles turned red and the ball continued moving downward. As mentioned before, if the participants’ paddles touched each other before the ball reached the interception axis, both paddles disintegrated, resulting in a failure to intercept the ball (i.e., unsuccessful interception). The occasional trials in which such a collision occurred after ball interception were considered successful as the common goal of intercepting the ball was achieved. Two seconds after ball arrival at the interception axis (regardless of a successful or unsuccessful interception), the paddles turned to their original white color and the translucent red rectangles would appear again for the team to start a new trial.

Balls moved downward following rectilinear trajectories and approached the interception axis under different angles. Similar to our previous studies (Benerink et al., 2016, 2018), the design included five standard ball departure positions (*Y* = +64 cm) and five standard arrival positions (*Y* = 0 cm), both at *X* = -42, -21, 0, +21, and +42 cm. Combining the five departure positions with the five arrival positions gave rise to a total of 25 standard trajectories. On each trial, a random distance between -10.5 cm and +10.5 cm was added to both the standard departure and arrival positions of the selected trajectory, shifting the entire trajectory to the left or right, while keeping trajectory incidence angle [or, equivalently, lateral ball movement (LBM) between the *X*-coordinates of ball departure and arrival positions] the same. This way, balls could appear and arrive anywhere between *X* = -52.5 cm and X = +52.5 cm (see **Figure 2**). In each block, all 25 trajectories appeared with two different vertical ball velocities resulting in a total of 50 fully randomized trials per block.

Both experimental doubles sessions (S2 and S3) started off with ten familiarization trials. Besides intercepting a number of balls, participants were asked to purposely miss one as well and to make contact with the other participant’s paddle, so as to experience all action possibilities, constraints and their outcome during these familiarization trials. In each doubles session, all teams completed four blocks consisting of 50 trials that were presented in random order. This resulted in a total of 200 trials for each team per doubles session, which took a team about an hour to complete.

## Results

### Interception Performance

**FIGURE 3:**
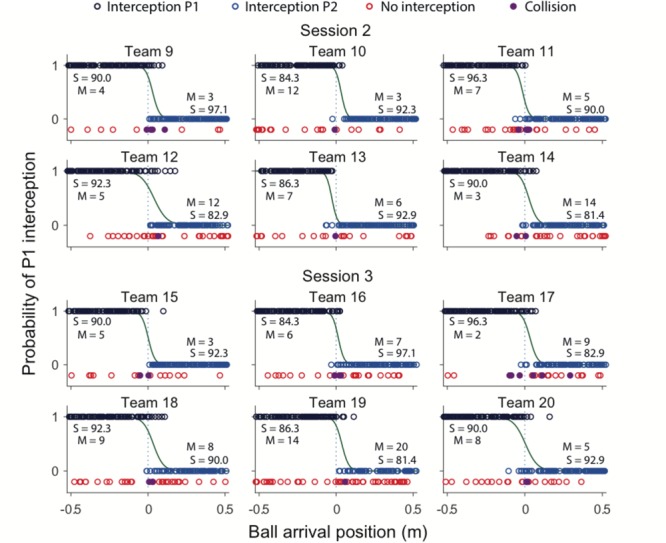
Graphical summary of interception performance as a function of ball arrival position for all 12 teams separately. Ball arrival positions for each successful trial are indicated by dark blue (P1 interception) and light blue (P2 interception) circles. Ball arrival positions of unsuccessful trials are indicated by red circles (errors) and purple dots (collisions). The green curves depict the logistic curves representing the probability that P1 (*p* = 1) or P2 (*p* = 0) will intercept the ball as a function of ball arrival position. The horizontal dashed gray lines at ball arrival position 0 cm indicate the center of the interception axis. For each team, S-scores from the individual sessions, as well number of misses (M) for each individual player in the BAP ranges outside ± 15 cm observed during the team sessions are indicated.

Team compositions (in terms of differences in individual S-scores) as well as their performances (in terms of percentage intercepted balls over all blocks) are presented in **Table 2**. Team performance varied between 76.0 and 94.0%, for an overall mean of 87.3 ± 4.3% (corresponding to a total of 2095 successful interceptions). Collisions leading to unsuccessful interception were rare, occurring in 1.3% (i.e., 31) of all 2400 trials. **Figure 3** provides a graphical summary of the interception results as a function of the ball’s arrival position on the interception axis for all 200 trials of each team separately. To this end, interceptions accomplished by the P1 (dark blue circles) and by the P2 (light blue circles) players were plotted on separate axes (corresponding to the probability of interception by the P1), allowing visual discrimination of who intercepted the balls where. Trials in which both participants failed to intercept the ball (red circles) and trials resulting in a collision between the participants’ paddles (purple circles) are also presented. As also observed in Benerink et al. (2016, 2018), collisions mainly occurred around the center of the interception axis, while misses were widely distributed over the interception axis.

### Boundary Location and Overlap Between Interception Domains

Largely corroborating the general observations of Benerink et al. (2016, 2018), inspection of **Figure 3** revealed a clearly visible but nevertheless somewhat fuzzy separation of interception domains for all teams. In a first step to assess the effect of within-team skill-level differences on the separation of interception domains, we followed the procedure adopted in Benerink et al. (2016, 2018) for determining the location of the boundary and the amount of overlap between interception domains. To this end, simple logistic probability curves for interception by P1 (*p* = 1) and P2 (*p* = 0) were derived for each team^2^, using ball arrival position (BAP) along the interception axis as a predictor (green lines in **Figure 3**). From the logistic regression equations, the location of the boundary between interception domains was calculated for each team as the location of the symmetry (*p* = 0.5) point and the amount overlap as the distance along the interception axis between the *p* = 0.05 and *p* = 0.95 points (see Cox and Snell, 1989). As can be seen from **Table 2**, boundary locations varied over teams between -2.8 and +3.9 cm, for an overall mean of 1.8 ± 2.2 cm. Interception domains of individual team members revealed overlaps varying between 10.2 and 24.8 cm for an overall mean of 14.9 ± 4.9 cm.

**FIGURE 4:**
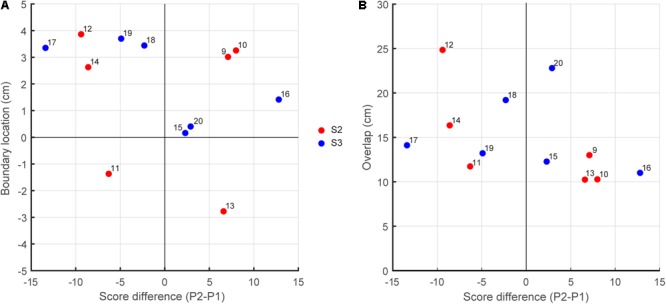
**(A)** Boundary location as a function the within-team skill-level differences. **(B)** Overlap between interception domains as a function the within-team skill-level differences.

We tested whether the better-skilled player took responsibility for a larger interception domain, resulting in a boundary location shifted from the center of the interception axis in the direction of lesser-skilled player. Contrary to this hypothesis, however, such a shift was not systematically observed in our data. Plotting the boundary location as a function the within-team skill-level differences (**Figure 4A**) did not reveal the expected association, with positive P2–P1 difference leading to a shift in boundary location to the left (points in the fourth quadrant of **Figure 4A**) and negative P2–P1 difference leading to a shift to the right (points in the second quadrant of **Figure 4A**). From the 12 teams studied, only six (three out of six in S2 and three out of six in S3) revealed boundary locations in the predicted quadrants. For the relatively homogeneous within-team skill-level differences in S2, the chance of finding a smaller (larger) interception domain for lesser-skilled (better-skilled) player was thus as large as finding a shift in the opposite direction. Even for the two teams with the largest skill-level differences (S3 teams 16 and 17; see **Table 2**) one did not reveal the expected behavior: Team 16 had a larger interception domain for the lesser-skilled player. Likewise, plotting the amount of overlap between interception domains as a function of the within-team skill-level differences (**Figure 4B**) did not reveal any systematic relation.

### GLMER Analysis

While within-team skill-level differences did not reveal systematic effects on the location of the boundary and the amount of overlap between interception domains, we noted that these global analyses were, for each team, based on the full set of ball trajectories presented. Yet, balls could not only arrive at different positions on the interception axis but could also arrive there with different speeds and different angles of approach (i.e., different amplitudes of LBM resulting from the combination of BAPs with different ball departure positions). In order to test whether within-team skill-level differences might indeed be observed for specific BSs and/or specific amplitudes of LBM, we extended the analysis to a generalized linear mixed effects regression (GLMER), using the glmer function from the lme4 package (Bates et al., 2015). In addition to within-team skill-level difference (P2–P1; see **Table 2**), potential predictors of the binary outcome (interception by P1 = 1 and by P2 = 0) were BAP, LBM, BS, and session (fixed effects). Team was included in the overall analysis as a random-effect variable.

We started out with a null model, which included only the effect of BAP (thereby comparable to the set of simple logistic regressions described above). Predictors were then added to the model in a stepwise forward manner, starting with the main predictors, followed by their two-way interactions. In order to avoid possible multicollinearity, predictors were not included in the model simultaneously if they showed high correlation (ρ > 0.7). Predictors were retained in the model if they turned out to be significant (α = 0.05) and simultaneously led to a decrease of the Akaike information criterion (AIC) of more than 2 (cf. Burnham and Anderson, 2004). This procedure was followed until no further improvement of the model could be achieved. Collinearity of the model was then reassessed on the basis of the variance inflation factor (VIF). If the VIF was above a threshold value of 3 (as suggested by Zuur et al., 2010), removal of the predictor from the model was considered.

Importantly, whereas adding LBM and BS to the original null model with BAP as predictor variable improved outcome prediction, this was not the case for the within-team skill-level difference and session^3^ variables; inclusion of within-team skill-level difference, either as a continuous or as a binary variable, did not significantly improve the prediction (either through a main effect or through an interaction with other variables) nor did it lead to the criterion reduction in AIC.^4^ We therefore conclude that, even for specific BSs and for specific ball trajectories, within-team skill-level differences did not systematically affect which player intercepted the ball where. Considering that, on the other hand, systematic effects of BAP, LPM, and BS were observed, our overall pattern of findings thus provides quite compelling evidence against a systematic role of within-team skill-level differences in the location of the division of interception space.

The final model included the fixed effects of BAP, LBM, BS, and the BAP x BS interaction effect, as well as a random effect of Team (see **Table 3**). While the (strong) effect of BAP was, of course, to be expected from observation of **Figure 3**, the others were not. First of all, the analysis demonstrated that the effect of BAP was moderated by BS. For balls moving at the lower speed, the probability curve was somewhat shallower than for balls moving at the higher speed, implying a larger degree of overlap between interception domains of the players when they had more time at their disposal. Second, the effect of LBM indicated that angle of approach to the BAP influenced which player intercepted the ball. This finding most likely reflects the so-called angle-of-approach effect observed in individual lateral interception tasks: balls arriving at the same position after the same motion duration give rise to kinematic interception patterns that vary systematically as a function of the incidence angle of the ball’s trajectory (Peper et al., 1994; Montagne et al., 1999; Michaels et al., 2006; Arzamarski et al., 2007; Ledouit et al., 2013).

**Table 3:** Generalized linear mixed model fit by maximum likelihood (Laplace approximation) for final model^a^.

Model variables						
**Random effects**	**Variance**	**Standard deviation**			**95 % CI lower**	**95 % CI upper**
Team (*n* = 12)	1.261	1.123			0.450	2.033
**Fixed effects**	**Estimate (β)**	**Standard error**	***p*-Value**		**95 % CI lower**	**95 % CI upper**
(Intercept)	1.173	0.429	0.006	^∗∗^	0.089	1.805
BAP	–52.130	5.646	<0.001	^∗∗∗^	–60.770	–35.682
Speed (2)	–0.933	0.428	0.029	^∗^	–1.896	0.016
LBM	7.076	0.978	<0.001	^∗∗∗^	4.211	8.683
BAP × speed (2)	–33.199	9.737	<0.001	^∗∗∗^	–49.625	–3.103

*Significance codes: ^∗∗∗^<0.001; ^∗∗^<0.01; ^∗^<0.05. CIs were calculated using parametric bootstrapping (*n* = 1000). ^*a*^Model formula in R-notation: result ∼ BAP + speed + LBM + BAP × speed + (1 | team).*

**FIGURE 5:**
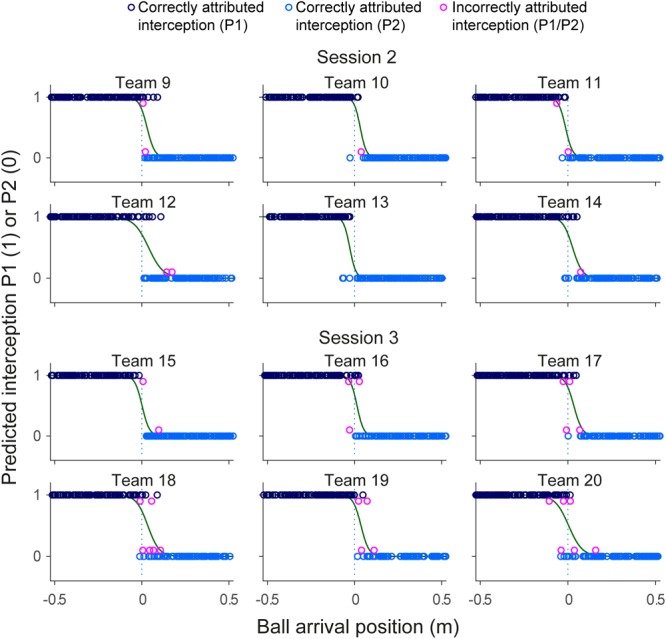
Graphical summary of interception performance predicted by the statistical GLMER model as a function of ball arrival position for all 12 teams separately. Ball arrival positions for correctly attributed interceptions are indicated by dark blue (P1 interception) and light blue (P2 interception) circles. Ball arrival positions of incorrectly attributed interceptions are indicated by pink circles with a slight vertical offset. The green curves depict the logistic curves representing the probability that LP (*p* = 1) or RP (*p* = 0) will intercept the ball as a function of ball arrival position. The horizontal dashed gray lines at ball arrival position 0 cm indicate the center of the interception axis.

**FIGURE 6:**
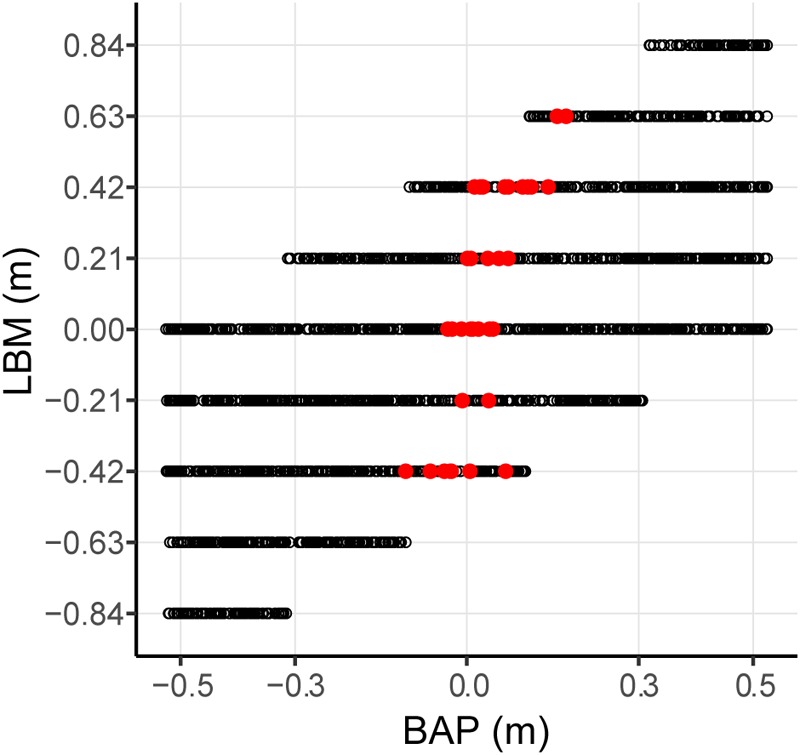
Effect of lateral ball movement (LBM) and ball arrival positon (BAP) combinations on GLMER model predictions for the total of 2095 intercepted trials. Red dots represent trials with incorrect predictions of which player intercepted the ball. Black dots represent trials with correct predictions of which player intercepted the ball.

Evaluation of the statistical pertinence of the GLMER model by a trial-by-trial examination of its predictions revealed that it correctly predicted interception by P1 or P2 in 98.4% of all successfully intercepted trials. In other words, of all 2095 trials resulting in interception, the GLMER-based model provided an incorrect prediction of who intercepted the ball in (only) 33 cases. As can be seen from **Figure 5**, the GLMER prediction errors (red circles) generally concerned balls arriving close to center, with a mean BAP of 3.4 ± 6.3 cm. **Figure 6** allows appreciating the supplementary effect of LBM, with direction (positive or negative) and magnitude of LBM revealing a relation with BAP of incorrectly predicted interceptions [correlation between LBM and BAP for prediction errors: *r*(31) = 0.75, *p* < 0.001].

Analysis of the GLMER model and its predictions of who intercepts which ball indicated that, while overall the correct prediction rate was very high, it required inclusion of the LBM variable. We will discuss the consequences hereof further on, but first move on to evaluate the action-based model of continuous interaction proposed by Benerink et al. (2016) to explain who intercepts which ball.

### Action-Based Model of Continuous Interaction

**FIGURE 7:**
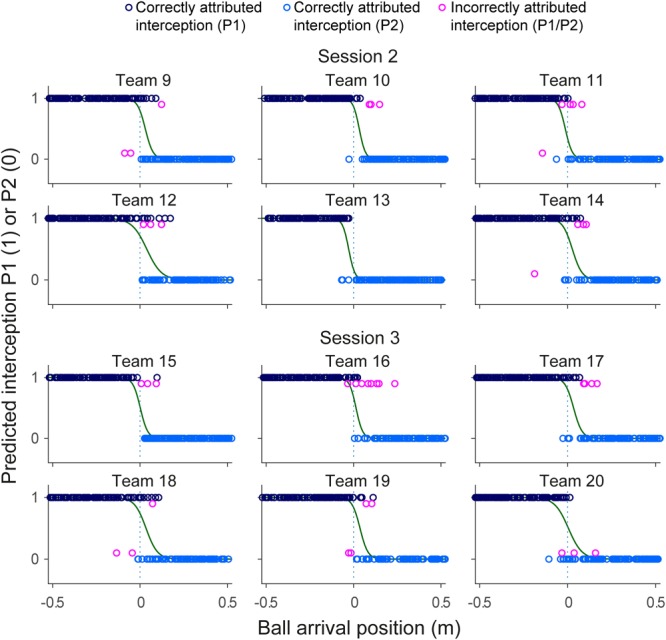
Graphical summary of interception performance predicted by the action-based model predicted as a function of ball arrival position for all 12 teams separately. Ball arrival positions for correctly attributed interceptions are indicated by dark blue (P1 interception) and light blue (P2 interception) circles. Ball arrival positions of incorrectly attributed interceptions are indicated by pink circles with a slight vertical offset. The green curves depict the logistic curves representing the probability that LP (*p* = 1) or RP (*p* = 0) will intercept the ball as a function of ball arrival position. The horizontal dashed gray lines at ball arrival position 0 cm indicate the center of the interception axis.

While the results of the present study did not reveal systematic effects of within-team skill-level differences, a separation of interception domains with a more or less fuzzy boundary was observed in all 12 teams. Benerink et al. (2016) suggested that, rather than being somehow predefined, such a separation in fact emerged from a continuous information-based interaction between the team members. More precisely, they suggested that this interaction was captured by the rates of change of angles β (see **Figure 1**). With both team members potentially engaging in interception for each ball, the one that first reaches a positive *d*β/*dt* (indicating that the ongoing interceptive movement is expedient) will be the one that intercepts the ball (see Benerink et al., 2016, 2018 for further details). When applied to the present data set, in its simplest form, the continuous interaction model correctly predicted the results in 97.8% of all successfully intercepted trials (i.e., for 2049 of the 2095 trials concerned, with 46 erroneous predictions). Analogously to **Figures 5, 7** presents the predictions of the continuous interaction model, and their correctness compared to the measured outcome of the trials, for each team separately. In visualizing these results, it is important to bear in mind that, contrary to the GLMER analysis, the continuous interaction model was not fitted to the observed results: the resulting (fuzzy) separation of interception domains shown in **Figure 7** is a consequence of the between-player interaction prior to interception.

**FIGURE 8:**
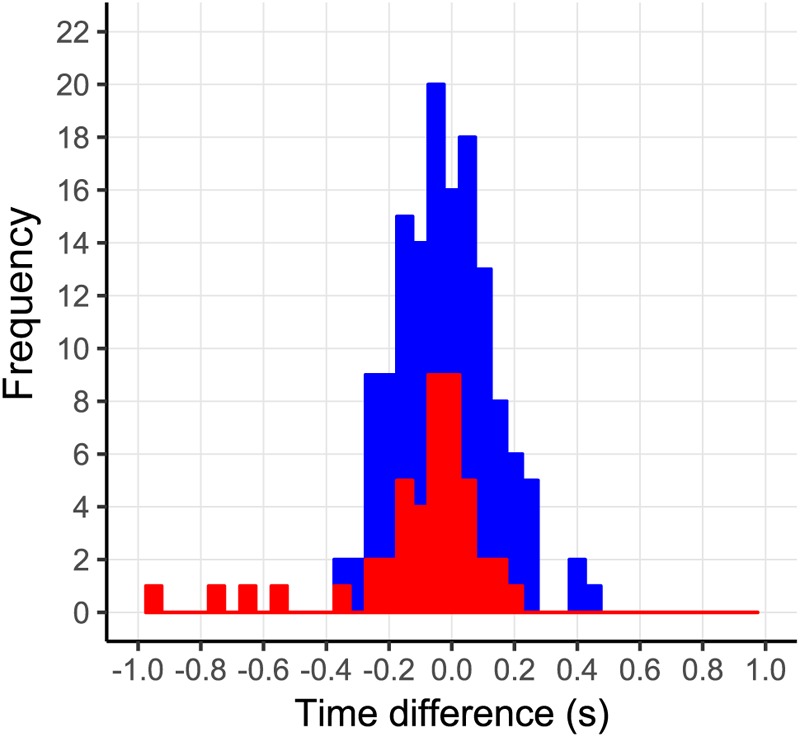
Time-of-occurrence differences (P2–P1) for first instances of *d*β/*dt* > 0. All trials in which both players reached *d*β/*dt* > 0 are shown in blue. Trials in which both players reached *d*β/*dt* > 0 that were incorrectly predicted by the action-based model are shown in red.

**FIGURE 9:**
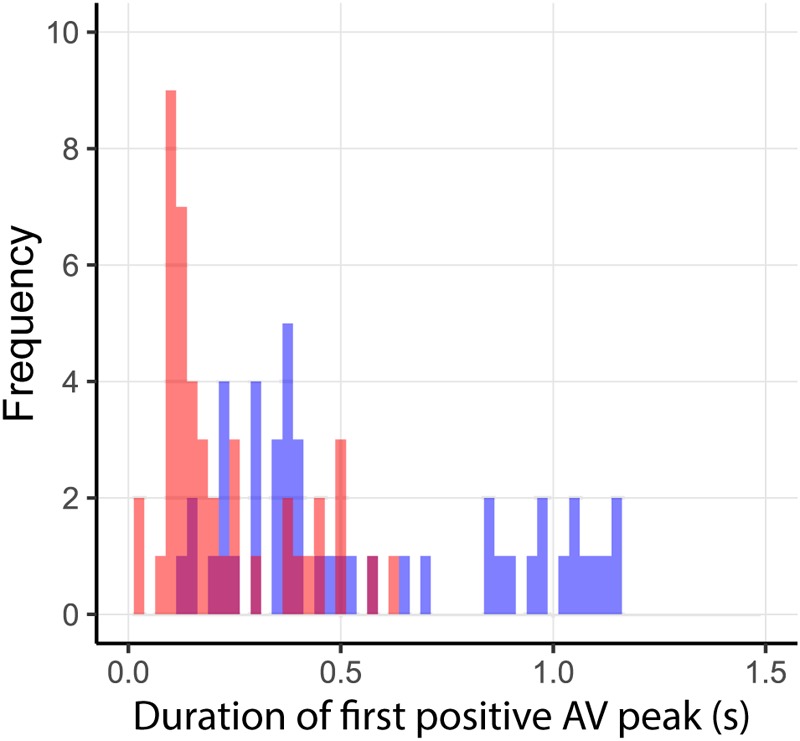
Duration of first *d*β/*dt* > 0 period for incorrect predictions by the action-based model. Durations of *d*β/*dt* > 0 for intercepting and non-intercepting players are indicated in blue and red, respectively.

Incorrect prediction by the action-based model of who will intercept which ball occurs when the non-intercepting player is the one who reaches positive *d*β/*dt* first. Trials in which both players reached positive *d*β/*dt* occurred in 144 of the 2095 successfully intercepted trials, of which 46 resulted in incorrect prediction of who intercepted the ball. As can be seen from **Figure 8**, in almost all these trials, both team members reached positive *d*β/*dt* at approximately the same moment (i.e., within 200 ms from each other), implying that they hardly had time to adjust their behavior to that of their team mate. Moreover, as can be seen from **Figure 9**, in these trials, one of the team members often maintained the state of positive *d*β/*dt* for only a short (<200 ms) duration; that is, they did in fact not pursue their interceptive movement in an expedient way. Enriching the criterion for attributing the interception to a given team member by selecting the team member that first reached positive *d*β/*dt* and maintained it for at least 200 ms gave rise to correct predictions of who intercepted the ball in 99.1% of the 2095 successfully intercepted trials, leaving a mere 19 trials with incorrect predictions. We emphasize that our goal in developing the action-based model of continuous interaction to a certain extent here (by including a supplementary criterion) is not necessarily intended to be taken as a proposal for durably refining it (as it may lose its attractive parsimony when additional criteria are added), but to demonstrate that it is capable of explaining, solely on the basis of the informational dynamics of the two-paddle-and-ball system, which team member will pursue the interception attempt and which will abandon it.

## Discussion

The current study was designed to investigate the effects of skill-level differences between team members on how the doubles-pong task is performed. Replicating earlier findings (Benerink et al., 2016, 2018), all teams showed a distinct but fuzzy boundary between interception domains. From an account of shared understanding of a tacitly agreed-upon boundary as a basis for assigning the interception to either player (i.e., the left and right player take responsibility for balls that will arrive left or right of the boundary, respectively), we expected that the boundary would be closer to the lesser-skilled player. As observed for the specific team in the Benerink et al. (2016) study that was characterized by large differences in individual skill levels, the hypothesis was that the better-skilled player would cover a larger interception domain. No matter how we analyzed the data (performing logistic-regression analyses separately for each team – that is, applying the methods that we used before in the Benerink et al., 2016, 2018 studies – or using linear mixed-effects logistic regression, controlling for potential unanticipated effects of other variables that were part of the design), we did not find any systematic effect of within-team skill-level differences on the location of the boundary. Rather, the GLMER analyses indicated that other factors, such as BS and the lateral movement of the ball, affected the division of interception space between the two team members. The GLMER model was able to correctly predict the player who intercepted the ball in about 98% of all successful trials. We also considered the action-based model of continuous interaction introduced by Benerink et al. (2016), in which the prediction of the division of labor between the players is based on the first player to be in a situation that affords interception, as specified by a zero-rate of change in participant-related base angle β. This model also predicted about 98% of who of the players made the successful interception.

Although of similar predictive power, the two models represent two diagonally different accounts. The GLMER model is a statistical model that was fit to the data a posteriori (i.e., which player intercepted the ball was used as an input variable to derive the model), optimizing a fair amount of degrees of freedom. The underlying logic of this model fits with an account in which the players base their decisions on who of the two will make an interception on an *a priori* boundary. In contrast, the action-based model goes with an account in which the boundaries can be identified *a posteriori* (i.e., the boundary and its characteristics emerge from the dynamics of the ball–player–player triad) but observed coordination patterns are predicted from *a priori* principles (cf. Benerink et al., 2016), without recourse to any form of data fitting.

As mentioned before, one account of how the two players each intercept their specific subset of balls is that they choose who will take which ball using a tacitly agreed boundary dividing the interception space. The agreement must be tacit because in the present experiments, players were not allowed to communicate other than through moving their paddle on the screen. In this account, presumably then, they arrive at such shared knowledge (e.g., Vesper et al., 2017) from interactions early on in their team session. For each approaching ball, the players have to determine on which side of the boundary it will pass and base their shared decision on this information. The statistical GLMER model that we built to account for the present data indicated that not only the BAP but also the way how the ball arrived at this position – the LBM and BS – affected the division of labor. Particularly, the factor of LBM is interesting, because it is indicative of an angle-of-approach effect in joint interception. When we translate these results to an account of joint decisions based on which side of the boundary a ball will pass, the predictions involved in these decisions will have to take many factors (including BS and angle of approach ball) into account. Interestingly, in individual lateral interception, the angle-of-approach effect has been reported repeatedly and has been taken to imply that an interceptive movement is not controlled toward a predicted future arrival position (e.g., Peper et al., 1994; Montagne et al., 1999; Dessing et al., 2002; Michaels et al., 2006; Ledouit et al., 2013). Thus, invoking an explanation relying on the prediction of a future BAP in joint interception seems problematic.

The alternative to predictive control in lateral interception is the use of continuous prospective information (e.g., Bootsma et al., 2016). A zero rate of change of angle β in the pong task qualifies as prospective information because upcoming interception is specified for current ball and paddle movement. The action-based model proposed by Benerink et al. (2016) and tested in the present study (see also Benerink et al., 2018) capitalizes on the use of this informational variable. Saying that the rate of change of angle β has reached zero boils down to saying that paddle movement is such that successful interception is forthcoming (if current conditions persist). In other words, a zero rate of change of angle β specifies expediency of current movement (cf. Benerink et al., 2016). When one of the two players is moving in such a way that s/he has reached a zero rate of change of β, the other player can (and should) stop moving and leave interception to the teammate. As mentioned before, this model accounted for about 98% correct predictions of the intercepting player. When we inspected the trials with incorrect predictions, we noted that in many of these cases both players reached a zero rate of change of β at about the same time and/or that the rate of change of β remained above zero for only a fraction of a second. Some straightforward fine-tuning the model to deal with these spurious results led to an almost perfect prediction of the intercepting player. This is not to suggest that elaborating the model toward better prediction should be the goal, but more to show how an action-based account seems to capture the phenomena very well without losing the elegance of its simplicity.

The doubles-pong task is an instance of the many ways in which two persons work together to attain a shared goal. As demonstrated in the current study, as well as in previous doubles-pong studies (Benerink et al., 2016, 2018), the two players in this task appear to have divided up interception space (with a fuzzy boundary), each taking care of a subset of the approaching balls. We argue that explaining this division of labor as emerging from the dynamics of the player–player–ball system leads to a more parsimonious account than one in which the players explicitly use a tacitly agreed-on boundary for deciding the player to take a specific ball. Previous studies also showed how different roles that members of dyads might take up emerge from the dynamics of the task. For instance, Davis et al. (2017) had two players coordinate two circular avatars (of different size), controlled by hand movements and presented on a shared screen. The stability of the balance of the players was manipulated by having them either stand with a normal base of support or in a heel-to-toe tandem stance. The balance manipulations led to one player taking on the role of leader and the other that of follower, without any instruction to do so. Another example comes from Richardson et al.’s (2015) study on an interpersonal collision-avoidance task. Here, two members of a dyad were to cyclically move a pointer between two targets on a shared screen. The targets were positioned on the corners of an invisible square, and each player was to move along one of the two diagonals. The instruction was to have the pointers not collide. Meeting task requirements, in theory, could have been realized in many ways, but the dyads all turned out to show a solution in which one dyad member moved along a straight path and the other along an elliptical path, while synchronizing target contacts. A final example involves dyads that have to perform a reciprocal aiming task (a Fitts’ task), either unimanually between two targets (as studied most often), bimanually with one hand moving the pointer and the other moving the set of targets, or in a dyad with one member of the dyad moving the pointer and the other moving the set of targets (Mottet et al., 2001). Interestingly, when allowed, people did move the set of targets, and when considering relative movements (i.e., pointer with respect to targets), the movement patterns essentially were the same across these three conditions. What is common in all these examples is that roles were not prescribed to the participants, but rather emerged from the dynamics given the task constraints (see also Riley et al., 2011). What sets apart the doubles-pong task, though, is that successful performance requires interception by only one of the two dyad members while the other member’s movement has to be such that no collision occurs. That is to say, whereas in the other tasks, both members of the dyad continuously interact in attempting to meet the common task goals, the doubles-pong task more resembles the emergence of discrete decisions.

## Conclusion

All in all, the aim of the present study was to investigate the effects of skill difference between the two participants in the doubles-pong task on the division of interception space. The results of our analysis did not suggest the presence of any straightforward effect of skill difference. Since we did not question our participants on this, we cannot say whether skill-level differences were consciously perceived by the players. Still, the boundaries between interception domains varied over teams. Whereas these boundaries were mostly halfway in-between the initial positions of the players’ paddles in the Benerink et al. (2016) study, there was considerably more variability among teams in the present study (see also Benerink et al., 2018). Perhaps, the asymmetries (see Lagarde, 2013) introduced by the skill differences in the present study did have an effect on the emerging patterns. However these asymmetries played out, the action-based account was able to capture the observed patterns. These patterns emerged from the informational couplings among the players and their environment (e.g., Mottet et al., 2001; Richardson et al., 2015; Davis et al., 2017; Nalepka et al., 2017), coordinating under the constraint of not colliding (see also Richardson et al., 2015). Although we cannot totally rule out an account based on shared knowledge of a boundary and the ability to correctly predict BAP, this action-based account seems the most parsimonious, and, thus, most promising to us.

## Author Contributions

NB, RC, FZ, and RB conceived and designed the study. NB ran the study. AvO, NB, FZ, and RB analyzed and interpreted the data. All authors participated in drafting the work and/or revising it critically for important intellectual content. The final version submitted was approved by all authors.

## Conflict of Interest Statement

The authors declare that the research was conducted in the absence of any commercial or financial relationships that could be construed as a potential conflict of interest.

## Footnotes

1 The sixth team of Benerink et al. (2016), with an individual performance level difference of 10.0%, revealed the idiosyncratic team behavior mentioned in the introduction and interpreted as potentially due to differences in skill level between its members.

2 All statistical analyses were performed in *R* version 3.4.3 (https://www.r-project.org/).

3 For the session variable, we note that adding BAP × session and LBM × session interaction effects did lead to a significant improvement of the model performance, but at the cost of increased VIF values. For this reason (i.e., to avoid collinearity), these two interaction effects were not included in the final model.

4 To explore the option that players when acting as team members showed different skill levels than when acting alone (i.e., in S1), we determined for each participant in both S2 and S3 (i.e., the team sessions) the number of misses in an area that was clearly to be covered by this specific participant. **Figure 3** gives the number of misses in the areas to the left or to the right of -15 and +15 cm for the P1 and P2, respectively. Although not directly comparable with the skill-level scores for S1 (because these scores were computed for players covering the full length of the interception axis), the number of misses provided an impression of individual skill levels during the team sessions, correlating significantly with skill level scores, *r*(22) = -0.72, *p* < 0.001. Next, we checked if these numbers of misses had any predictive value in the GLMER model. This turned out not to be the case.
